# Supplementary material for: Informational ecosystems partially explain differences in socioenvironmental conceptual associations between U.S. American racial groups
Source: Commun Psychol. 2025 Jan 20;3:5. doi: 10.1038/s44271-025-00186-w (PMC11747393; doi:10.1038/s44271-025-00186-w)
Supplement: Supplementary file 2 — Supplementary Information [file 44271_2025_186_MOESM2_ESM.pdf]

Supplementary Info

Supplementary Figure 1. Demographic and sample statistics. Of the total analyzed sample of 446 participants, 49.55% identified as Female, 47.76% identified as Black / African American. Of the 446 participants, 114 identified as White and Male, 119 as White and Female, 111 as Black and Male, and 102 as Black and Female. The mean age of the analyzed sample was 40.56 years (SD = 12.43; panel b). For Black and White identifying participants, the mean ages were 37.99 and 42.91, respectively, compared to the national averages of 36.3 and 44.2 (US Census, 2023). In this study, income was measured using bracket ranges, so direct comparison to national averages was not possible (panel b). To approximate the sample's income, counts were converted to the midpoint of each range (e.g., \$12,501–\$22,500 was recorded as \$17,500), or to the exact value for open-ended responses (e.g., 'below \$12,500' was recorded as \$12,500). Based on this method, Black and White participants had mean incomes of \$42,142.39 and \$44,285.65, respectively, compared to the national averages of \$32,360 and \$50,675 for individual earners (US Census, 2023). The association in our sample between Black identity and Left-leaning political affiliation, and White identity and Right-leaning political affiliation, aligns with trends found in previous research (Gilens, 2023). The 446 analyzed participants were recruited from 421 unique zip codes across 46 unique states within the United States. Histograms of Age and Income for the sample are shown below. Age was modestly correlated with Right-leaning news consumption, r(444) = 0.1, $p = 0.02$, 95%CI [0.01, 0.19]. News consumption was not correlated with either Income, r(444) = -0.04, $p = 0.233$, 95%CI [-0.13, 0.05] or Gender, r(444) = 0.04, $p = 0.214$, 95%CI [-0.05, 0.14].

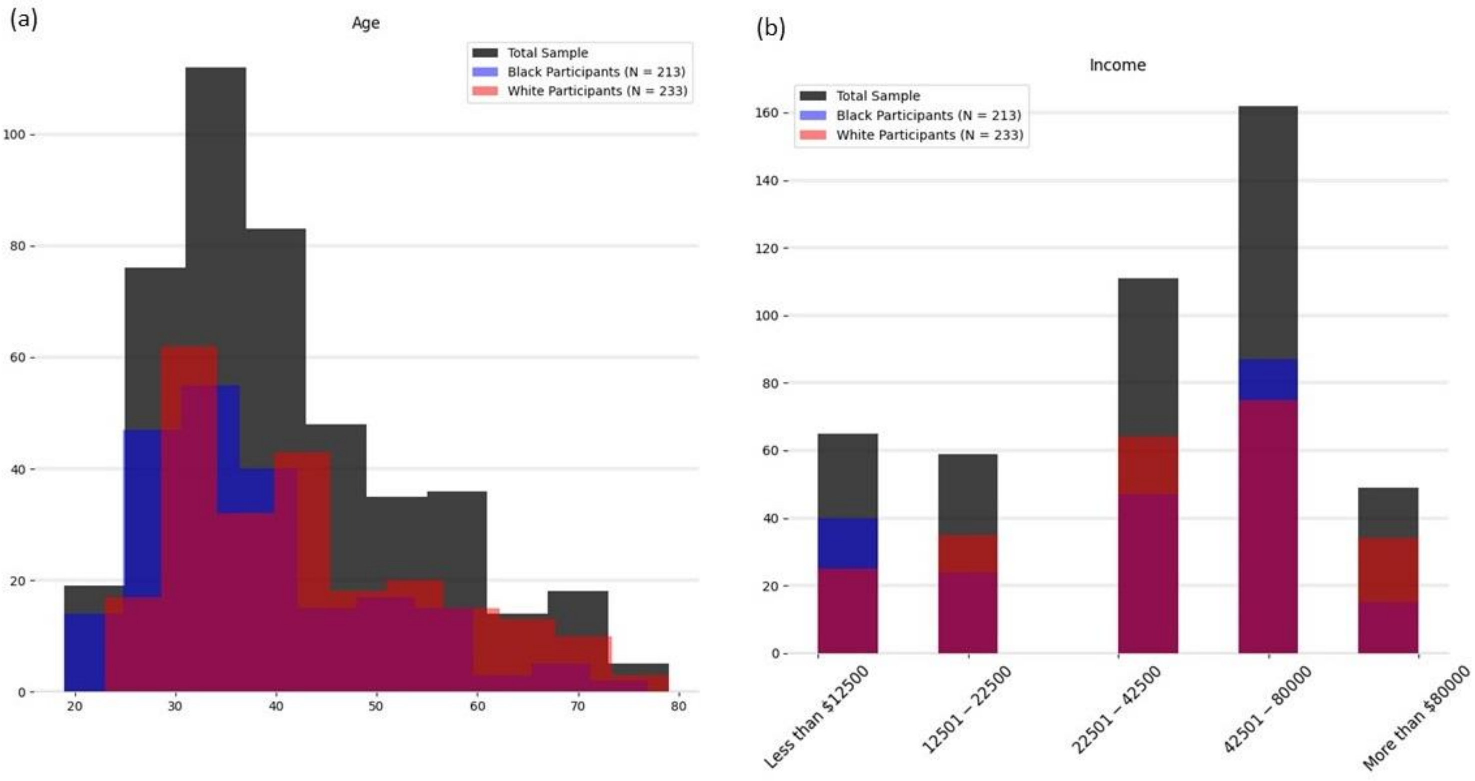

Supplementary Figure 2. Bar plot of similarity ratings for Left and Right news consumers along news differing concept pairs. Error bars mark 95% CI boundary. Associativity ratings are pool across Left and Right biased news consumers respectively. Left and right news consumers were defined as those whose mean news bias was above or below 3 (i.e., Center)

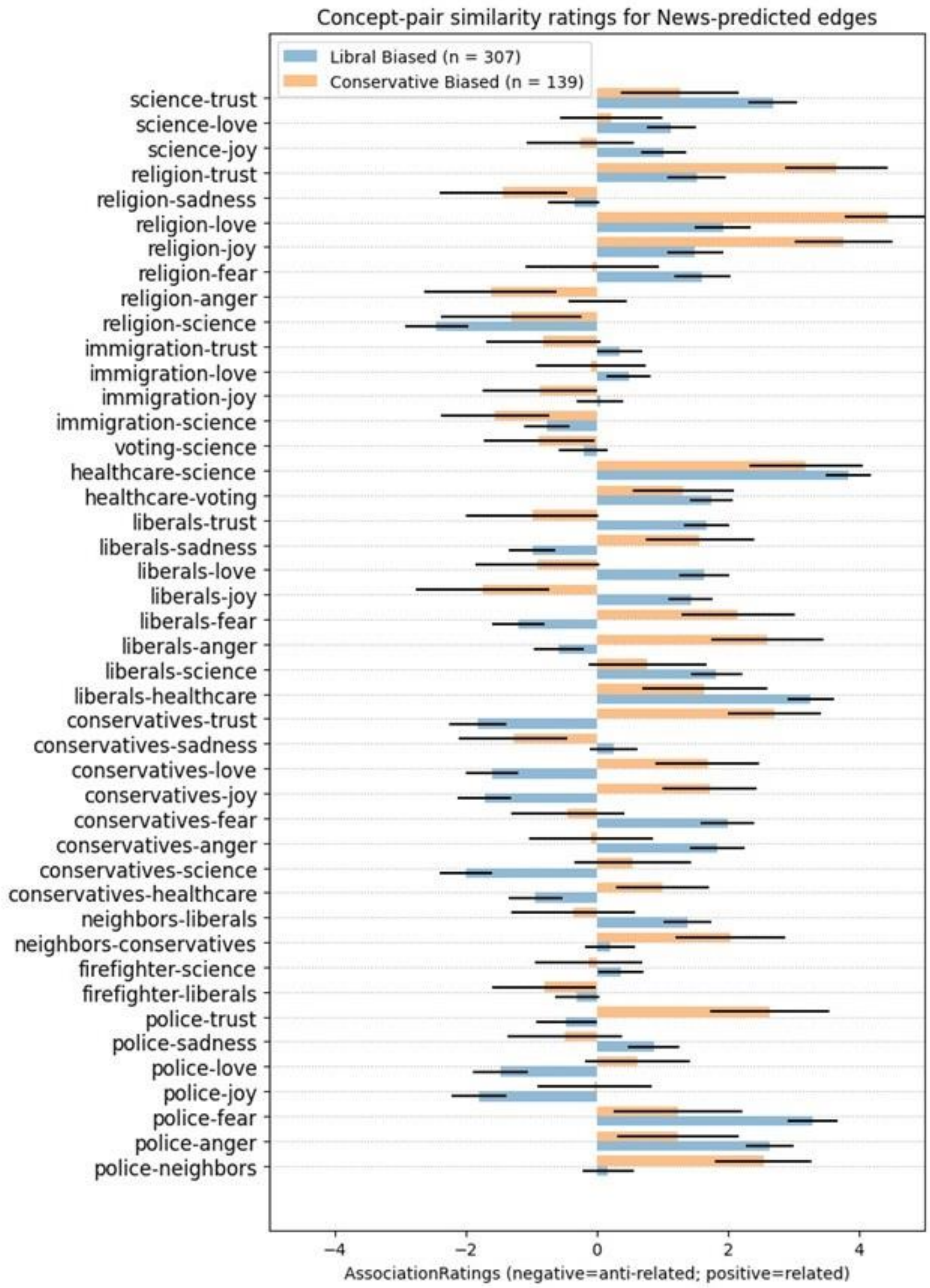

Supplementary Figure 3. Schematic of mediation model. In the mediation model, race was used to predict edge lengths between concept pair similarity, with news consumption as a mediator. The total effect is comprised of both direct and indirect effects. Partial mediation occurs when race predicted significant direct and indirect effects (through news consumption) on concept pair similarity.

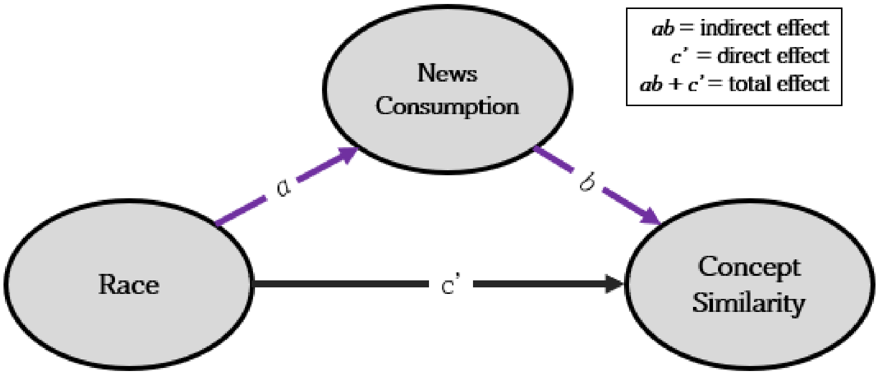

Supplementary Figure 4. Inter-partisan representational distance and News bias. To measure whether news bias effects were proportional with differences in concept pair representations across Left and Right news consumers. Concept-pair representational distance was computed as 1-r where r is the correlation of the distribution of -7 to +7 ratings for a given concept pair across Left- and Right- leaning news consumers (i.e., correlating rows from Figure 1b across Left and Right news consuming groups).

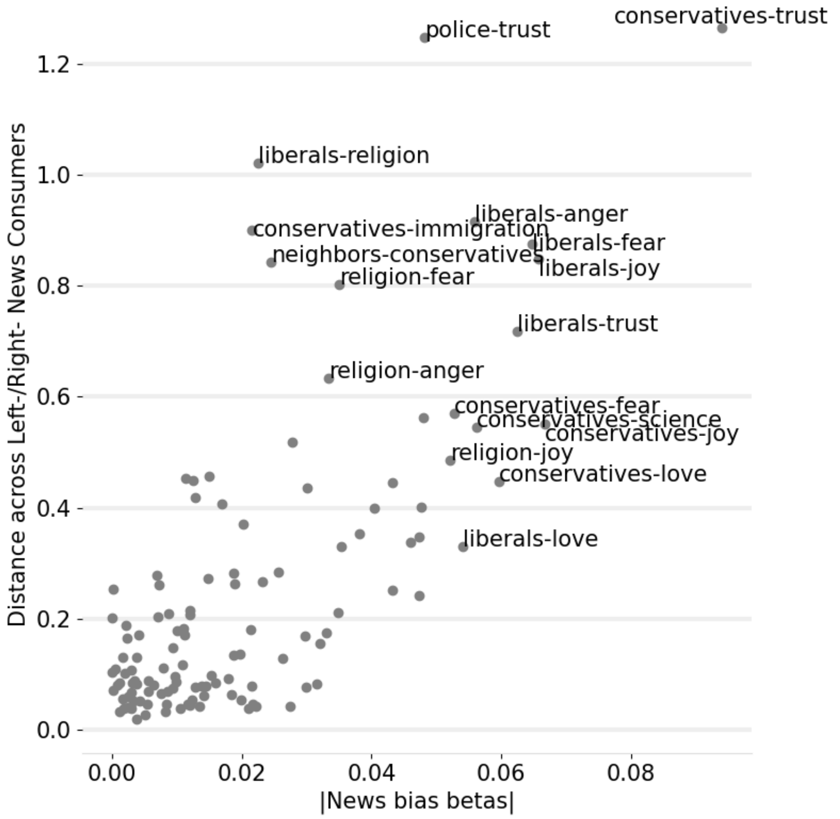

Supplementary Table 1. Table of complete regression model results. Predicted values are concept pair edge lengths; shorter lengths indicate stronger associations (more semantically relatedness) between concept pairs while longer lengths indicate the concept pair has weaker associations (less semantically relatedness). The news bias predictor is coded such that greater values communicate the consumption of more biased right-leaning news. For example, for the concept pair *police-trust*, an increase in right leaning news consumption is significantly associated with a shorter edge length (stronger association) between the concept pair. Significance was determined via a 10,000-iteration permutation test. Asterisks denote concept pairs that were significant with FDR correction for family-wise error. Although no differences were initially expected for the concept *firefighter*, several differences emerged. Notably, *firefighter-conservatives* and *police-firefighter* associations differed by race, while *firefighter-liberal* varied by news consumption. One explanation could be that White and Right-leaning participants may associate *firefighters* and *police* as belonging to the same group (i.e., public servants), whereas Black and Left-leaning participants may view them as distinct entities. This is further supported by the observation that *police* was generally associated with conservatism across all groups.

	β-Race (Black=1)	β-Gender (Female=1)	β-Income	β-Age	β-NewsBias
**police-firefighter**	0.056*	0.009	0.003	-0.001	-0.011
**police-neighbors**	0.043	-0.002	-0.010	0.000	-0.046*
**police-conservatives**	0.091*	0.031	0.000	0.002	0.002
**police-liberals**	-0.038	-0.009	0.003	0.001	0.013
**police-healthcare**	0.038	-0.004	-0.005	0.002	0.005
**police-voting**	-0.011	-0.007	-0.009	0.001	0.006
**police-immigration**	-0.001	0.009	-0.017	0.000	-0.009
**police-religion**	0.059*	-0.008	-0.006	0.000	0.003
**police-science**	0.001	0.031	-0.007	-0.001	-0.008
**police-anger**	-0.010	-0.033	0.003	0.002	0.030*
**police-fear**	-0.084*	-0.025	0.020	0.002	0.035*
**police-joy**	0.011	-0.004	-0.007	-0.002	-0.026*
**police-love**	0.033	-0.029	-0.015	0.000	-0.03*
**police-sadness**	-0.065*	0.005	0.009	0.000	0.026*
**police-trust**	0.076*	0.004	-0.012	-0.001	-0.048*
**firefighter-neighbors**	0.032	-0.002	-0.009	-0.002	0.003
**firefighter-conservatives**	0.075*	0.023	-0.015	0.000	-0.014
**firefighter-liberals**	-0.001	-0.023	-0.015	0.000	0.018*
**firefighter-healthcare**	0.000	-0.017	-0.017	0.001	-0.003
**firefighter-voting**	0.056*	0.013	-0.007	0.000	0.005
**firefighter-immigration**	0.030	0.001	0.000	0.001	0.011
**firefighter-religion**	0.016	0.004	-0.008	0.000	0.002
**firefighter-science**	0.056*	0.033	-0.005	0.000	0.022*
**firefighter-anger**	-0.010	-0.015	-0.004	-0.001	-0.001
**firefighter-fear**	-0.012	0.012	0.006	0.000	-0.019
**firefighter-joy**	-0.005	-0.022	0.005	0.001	-0.004
**firefighter-love**	0.021	-0.001	0.004	-0.001	0.001
**firefighter-sadness**	-0.003	-0.006	-0.008	-0.001	-0.009
**firefighter-trust**	0.010	-0.035	-0.002	-0.001	-0.006
**neighbors-conservatives**	0.111*	0.040	-0.022	0.001	-0.024*
**neighbors-liberals**	-0.046	-0.004	0.006	0.000	0.04*
**neighbors-healthcare**	0.012	0.017	-0.005	0.000	0.008
**neighbors-voting**	0.037	0.032	-0.004	0.000	0.006
**neighbors-immigration**	0.029	-0.017	-0.009	0.002	0.010
**neighbors-religion**	0.020	0.057	-0.007	-0.001	-0.007
**neighbors-science**	0.016	-0.021	-0.010	0.000	0.012
**neighbors-anger**	0.002	0.022	0.008	0.001	0.000
**neighbors-fear**	-0.016	0.017	-0.003	0.001	0.000
**neighbors-joy**	0.024	0.012	-0.006	0.002	0.003
**neighbors-love**	0.029	0.006	-0.004	0.001	0.008
**neighbors-sadness**	0.002	0.028	0.003	0.000	0.01
**neighbors-trust**	0.047	-0.016	-0.014	0.000	0.002
**conservatives-liberals**	-0.138*	0.001	0.012	0.002	-0.001
**conservatives-healthcare**	-0.058*	0.018	-0.018	-0.001	-0.035*
**conservatives-voting**	0.022	0.042	0.008	0.001	-0.012

**conservatives-immigration**	-0.013	0.062	0.006	0.002	-0.022
**conservatives-religion**	0.114*	0.050	-0.002	0.001	-0.004
**conservatives-science**	-0.062*	0.001	-0.006	0.000	-0.056*
**conservatives-anger**	0.074*	0.006	0.002	0.001	0.048*
**conservatives-fear**	-0.007	0.024	-0.020	0.001	0.053*
**conservatives-joy**	-0.036	0.034	-0.014	-0.001	-0.067*
**conservatives-love**	0.006	0.036	-0.006	0.000	-0.06*
**conservatives-sadness**	0.032	-0.034	0.010	0.001	0.032*
**conservatives-trust**	-0.025	0.019	-0.010	0.000	-0.094*
**liberals-healthcare**	0.067*	0.008	0.012	0.000	0.047*
**liberals-voting**	0.049	-0.014	-0.013	0.002	0.019
**liberals-immigration**	0.038	0.051	0.009	0.001	-0.011
**liberals-religion**	-0.078*	-0.041	0.001	0.000	0.023
**liberals-science**	0.127*	0.016	0.007	0.000	0.043*
**liberals-anger**	0.047	0.059	0.003	0.000	-0.056*
**liberals-fear**	0.006	0.056	-0.003	0.000	-0.065*
**liberals-joy**	-0.037	-0.036	-0.004	0.001	0.066*
**liberals-love**	0.008	-0.035	-0.009	0.000	0.054*
**liberals-sadness**	0.035	0.056	-0.007	0.000	-0.043*
**liberals-trust**	0.020	-0.028	0.001	0.001	0.062*
**healthcare-voting**	0.033	0.016	0.011	0.002	0.021*
**healthcare-immigration**	0.040	0.012	-0.010	0.000	0.011
**healthcare-religion**	-0.025	-0.008	-0.009	0.001	0.002
**healthcare-science**	0.032	0.000	-0.010	0.001	0.02*
**healthcare-anger**	-0.016	-0.011	-0.002	0.000	-0.001
**healthcare-fear**	0.032	0.010	0.000	0.000	0.004
**healthcare-joy**	-0.060*	0.000	-0.016	0.000	0.007
**healthcare-love**	0.010	-0.001	-0.006	0.001	0.014
**healthcare-sadness**	-0.003	0.007	0.009	-0.001	-0.004
**healthcare-trust**	-0.002	-0.020	-0.008	0.001	-0.002
**voting-immigration**	0.030	0.009	-0.006	0.002	0.000
**voting-religion**	0.001	0.035	-0.004	0.001	-0.008
**voting-science**	0.029	0.003	0.011	-0.001	0.022*
**voting-anger**	-0.011	0.019	-0.004	0.002	-0.019
**voting-fear**	-0.013	0.026	-0.001	0.002	-0.017
**voting-joy**	-0.033	-0.033	-0.020	-0.003*	0.014
**voting-love**	0.022	0.011	-0.003	0.000	0.003
**voting-sadness**	-0.008	0.000	0.001	0.000	-0.003
**voting-trust**	0.061*	0.032	-0.001	0.000	0.015
**immigration-religion**	0.017	-0.008	-0.006	-0.001	0.013
**immigration-science**	0.041	-0.021	0.010	-0.001	0.02*
**immigration-anger**	0.013	0.027	-0.013	-0.001	-0.020
**immigration-fear**	-0.012	0.028	-0.010	-0.001	-0.015
**immigration-joy**	-0.018	-0.022	-0.013	0.000	0.023*
**immigration-love**	0.005	-0.042	-0.008	-0.001	0.022*
**immigration-sadness**	-0.034	-0.006	-0.005	0.000	-0.013
**immigration-trust**	-0.021	-0.035	-0.005	-0.002	0.033*
**religion-science**	-0.117*	-0.043	-0.004	0.001	-0.03*
**religion-anger**	0.064*	0.003	0.001	0.002	0.033*
**religion-fear**	0.058	0.034	0.007	0.002	0.035*
**religion-joy**	-0.109*	-0.001	-0.006	-0.001	-0.052*
**religion-love**	-0.062*	-0.004	-0.013	-0.001	-0.047*
**religion-sadness**	0.065*	0.026	0.004	0.000	0.028*
**religion-trust**	-0.077*	-0.003	-0.008	-0.001	-0.048*
**science-anger**	-0.041	-0.004	-0.011	0.000	-0.009
**science-fear**	-0.063*	-0.028	-0.024	0.001	-0.021
**science-joy**	0.034	0.001	-0.002	0.000	0.032*
**science-love**	0.054*	-0.011	0.004	0.000	0.028*
**science-sadness**	-0.028	-0.002	0.004	-0.001	-0.004
**science-trust**	0.071*	0.026	-0.008	0.002	0.038*
**anger-fear**	0.053	0.010	-0.012	0.000	0.002
**anger-joy**	-0.070*	0.000	-0.017	0.000	-0.012
**anger-love**	-0.065	0.019	-0.005	0.000	-0.016
**anger-sadness**	0.019	-0.011	0.002	0.000	0.000
**anger-trust**	-0.054	0.005	-0.012	-0.001	-0.007

**fear-joy**	-0.052	0.003	-0.003	-0.001	-0.018
**fear-love**	-0.054	-0.028	-0.005	0.000	-0.015
**fear-sadness**	0.009	-0.017	0.001	0.000	-0.011
**fear-trust**	-0.047	-0.011	-0.013	0.000	-0.014
**joy-love**	-0.007	-0.006	0.011	-0.001	0.003
**joy-sadness**	-0.080*	0.029	-0.008	0.000	-0.012
**joy-trust**	0.024	-0.017	0.005	0.000	-0.010
**love-sadness**	-0.056	0.014	0.007	0.000	-0.012
**love-trust**	0.012	-0.015	0.012	0.001	0.004
**sadness-trust**	-0.039	-0.010	-0.024	0.000	-0.009

Supplementary Table 2. Full Mediation model results. For the direct effect of Race positive weights denote Black Identity; For the indirect effect of News Consumption, positive values denote liberal news consumption. All but three of the edges that differ by Race maintained a direct effect on concept pair edge. That is, News Consumption explained differences observed by Race for three concept pairs, namely, *conservative-healthcare, religion-anger,* and *religion-love***.** Significance was determined via a 10,000-iteration permutation test. Asterisks denote concept pairs that were significant with FDR correction for family-wise error.

	Total Pathway	Direct Pathway (Race)	Indirect Pathway (News Consump.)	Sobel Test
**police-fear**	-0.113*	-0.096*	-0.017*	-0.31*
**police-sadness**	-0.081*	-0.069*	-0.012*	-0.22*
**police-trust**	0.106*	0.083*	0.023*	0.42*
**firefighter-science**	0.048	0.058*	-0.010*	-0.18*
**neighbors-conservatives**	0.122*	0.111*	0.011*	0.21*
**conservatives-healthcare**	-0.036	-0.052	0.017*	0.31*
**conservatives-science**	-0.033	-0.060*	0.027*	0.49*
**conservatives-anger**	0.044	0.068*	-0.023*	-0.42*
**liberals-healthcare**	0.040	0.062*	-0.022*	-0.41*
**liberals-science**	0.103*	0.123*	-0.020*	-0.37*
**religion-science**	-0.107*	-0.121*	0.013*	0.24*
**religion-anger**	0.038	0.054	-0.017*	-0.30*
**religion-joy**	-0.078*	-0.103*	0.025*	0.46*
**religion-love**	-0.030	-0.053	0.023*	0.42*
**religion-sadness**	0.049	0.062*	-0.013*	-0.24*
**religion-trust**	-0.049	-0.072*	0.023*	0.42*
**science-love**	0.039	0.052*	-0.013*	-0.24*
**science-trust**	0.043	0.061*	-0.019*	-0.34*

Supplementary References

Gilens, M. (2023). Race, gender, and partisan politics in the United States. *Proceedings of the National Academy of Sciences*, *120*(25), e2307714120. https://doi.org/10.1073/pnas.2307714120

U.S. Census Bureau, U.S. Department of Commerce. (2023). Selected Population Profile in the United States. American Community Survey, ACS 1-Year Estimates Selected Population Profiles, Table S0201. Retrieved September 26, 2024, from [https://data.census.gov/table/ACSSPP1Y2023.S0201?q=United States&t=-0C&y=2023.](https://data.census.gov/table/ACSSPP1Y2023.S0201?q=United%20States&t=-0C&y=2023)
