# Supplementary material for: Informational ecosystems partially explain differences in socioenvironmental conceptual associations between U.S. American racial groups
Source: Commun Psychol. 2025 Jan 20;3:5. doi: 10.1038/s44271-025-00186-w (PMC11747393; doi:10.1038/s44271-025-00186-w)
Supplement: Supplementary file 3 — Reporting Summary [file 44271_2025_186_MOESM3_ESM.pdf]

nature portfolio

Corresponding author(s): Roberto Vargas

Last updated by author(s): Dec 20, 2024

Reporting Summary

Nature Portfolio wishes to improve the reproducibility of the work that we publish. This form provides structure for consistency and transparency in reporting. For further information on Nature Portfolio policies, see our Editorial Policies and the Editorial Policy Checklist.

Statistics

For all statistical analyses, confirm that the following items are present in the figure legend, table legend, main text, or Methods section.

n/a	Confirmed	
☐	☒	The exact sample size (*n*) for each experimental group/condition, given as a discrete number and unit of measurement
☐	☒	A statement on whether measurements were taken from distinct samples or whether the same sample was measured repeatedly
☐	☒	The statistical test(s) used AND whether they are one- or two-sided<br>*Only common tests should be described solely by name; describe more complex techniques in the Methods section.*
☐	☒	A description of all covariates tested
☐	☒	A description of any assumptions or corrections, such as tests of normality and adjustment for multiple comparisons
☐	☒	A full description of the statistical parameters including central tendency (e.g. means) or other basic estimates (e.g. regression coefficient) AND variation (e.g. standard deviation) or associated estimates of uncertainty (e.g. confidence intervals)
☐	☒	For null hypothesis testing, the test statistic (e.g. F, t, r) with confidence intervals, effect sizes, degrees of freedom and P value noted<br>*Give P values as exact values whenever suitable.*
☒	☐	For Bayesian analysis, information on the choice of priors and Markov chain Monte Carlo settings
☒	☐	For hierarchical and complex designs, identification of the appropriate level for tests and full reporting of outcomes
☐	☒	Estimates of effect sizes (e.g. Cohen's *d*, Pearson's *r*), indicating how they were calculated

Our web collection on statistics for biologists contains articles on many of the points above.

Software and code

Policy information about availability of computer code

Data collection

Study session and tasks were developed using Gorilla Experiment Builder, a web-based experiment development toolbox. A study session link generated from Gorilla was used to recruit participants via Mechanical Turk and Cloud Research Connect. The tasks and questionnaires can all be cloned and shared through Gorilla upon request from the corresponding author.

Data analysis

Data analyses were computed using openly available Python-compatible libraries within a Jupyter notebook. The codebook is formatted to be run on Google Colab thus requiring no locally installed software to reproduce. For a complete list of libraries used, please refer to the codebook in the provided GitHub link for the project (see data sharing statement).

For measures of political bias, AllSides provided a lookup table of their ordinal bias ranking for individual news sources (which was provided on 05/05/2023). Participant responses and corresponding bias look-ups are available via the the project GitHub (see data sharing statement). Bias rankings given to individual news sources are openly verifiable at https://www.allsides.com/media-bias/ratings.

For manuscripts utilizing custom algorithms or software that are central to the research but not yet described in published literature, software must be made available to editors and reviewers. We strongly encourage code deposition in a community repository (e.g. GitHub). See the Nature Portfolio guidelines for submitting code & software for further information.

Data

Policy information about availability of data

All manuscripts must include a data availability statement. This statement should provide the following information, where applicable:

- Accession codes, unique identifiers, or web links for publicly available datasets
- A description of any restrictions on data availability
- For clinical datasets or third party data, please ensure that the statement adheres to our policy

All de-identified data and code (in the form of a comprehensive Jupyter codebook) are openly available via corresponding authors' lab GitHub: https://github.com/ExCaLBBR/ExCaLBBR_Projects/tree/main/SocioenvironmentalGeometry

Human research participants

Policy information about studies involving human research participants and Sex and Gender in Research.

Reporting on sex and gender	Throughout the manuscript we were careful to refer to the study subsamples by their self-selected gender identities. During recruitment, participants were screened based on whether they "identified as either Male or Female genders" and whether they "identified as non-Hispanic White American or Black/African American". Recruitment queries through Mechanical Turk and Cloud Research Connect were structured such that equal proportions of these identity combinations were targeted for recruitment (i.e., 150 non-Hispanic White American Female identifying individuals; 150 Black/African American Female identifying individuals; 150 non-Hispanic White American Male identifying individuals; and 150 Black/African American Male identifying individuals).<br>Although the focus of the manuscript isn't gender, we included it as a covariate for critical analyses along with other identity variables such as age, income.
Population characteristics	The covariates used in critical analyses included gender (dummy coded such that Female = 1); income (ordinalized by income bracket (see SI2 for bracket breakdown)); and age. Although the specific location of the participant was not utilized in any analyses, the 446 analyzed participants were recruited from 421 unique zip codes across 46 unique states within the United States. The latter metric was to provide qualitative assurance that the sample did not overly represent a specific sociogeographical region within the United States.
Recruitment	Participants were recruited via Mechanical Turk and Cloud Research Connect. Because of administrative and technical complications with Mechanical Turk (MTurk), we transitioning from MTurk to Connect midway through data collection. There is a small chance that we may have recruited the same participant from both platforms. To catch if this had occurred, we queried participant's MTurk identifiers when they were recruited via Connect and cross references those identifiers with those previously collected from MTurk. No repeats were identified.
Ethics oversight	Carnegie Mellon University Institutional Review Board

Note that full information on the approval of the study protocol must also be provided in the manuscript.

Field-specific reporting

Please select the one below that is the best fit for your research. If you are not sure, read the appropriate sections before making your selection.

☐ Life sciences ☒ Behavioural & social sciences ☐ Ecological, evolutionary & environmental sciences

For a reference copy of the document with all sections, see nature.com/documents/nr-reporting-summary-flat.pdf

Behavioural & social sciences study design

All studies must disclose on these points even when the disclosure is negative.

Study description	We conducted a quantitative observational/survey study. All participants completed all parts of the study and no manipulations were conducted as part of the tasks or study session.
Research sample	Participants were recruited online in hopes of reflecting the general population within the United States. Although the specific location of participants were not utilized in any analyses, the 446 analyzed participants were recruited from 421 unique zip codes across 46 unique states within the United States. The latter metric was used to provide qualitative assurance that our sample did not overly represent a specific sociogeographical region within the United States. The complete demographic breakdown of the sample can be seen in SI2.
Sampling strategy	Stratified sampling was conducted such that equal proportions of the following groups were recruited: non-Hispanic White American Female identifying individuals; Black/African American Female identifying individuals; non-Hispanic White American Male identifying

individuals; and Black/African American Male identifying individuals. No sample size calculation was performed to determine recruitment target. The total sample recruitment target of 600 was the largest sample obtainable given resource constraints. We feel there is sufficient variability and representation for the covariates of interest including age, income, and partisan news consumption. Moreover, there was substantial variability in the sociogeographical location of the sample thus providing further converging support for the sample's representativeness.

Data collection	Study session and tasks were developed using using Gorilla Experiment Builder, a web-based experiment development toolbox. The link generated from Gorilla was then used to recruit participants via Mechanical Turk and Cloud Research Connect. Participants were required to complete the study session at a desktop computer or laptop (i.e., excluding participation using a phone or tablet). The relevant measures for the study session were sequentially collected in the following order: consent form; demographic intake; Pair-wise RAting Method (PRaM); news consumption questionnaire.
Timing	The complete sample was collected between February and March of 2023.
Data exclusions	A target sample of 600 participants was recruited via Amazon Mechanical Turk and Cloud Research Connect. Participants were included in the sample if they completed the study session (585 participants) identified as non-Hispanic White American or Black/African American (572 of the 585 participants), and as Male or Female genders (567 of the 572 participants). For news-based analyses, only participants who included news consumption responses which could be assessed for bias were included. This restriction resulted in a final sample of 446 participants (for complete demographic breakdown see SI2). Although we structured our recruitment queries to target specific populations, further investigation of participant responses to the demographic intake informed us that they were ineligible to be included in the analyses of target groups (e.g., self-identified gender as non-binary or identified as Hispanic White).
Non-participation	A total of 15 participants did not complete all necessary parts of the study session for inclusion. These participants did not complete the questionnaire asking participants about news consumption which was the final section of the study session. Given that participants' responses to this questionnaire are critical to the analyses presented in this manuscript, they were subsequently excluded. Participants either timed out of the study session or chose to terminate their existing session.
Randomization	Study sessions were structured such that all participants were expected to complete all parts of the study. Participants were collected in the four following batches: 150 non-Hispanic White American Female identifying individuals; 150 Black/African American Female identifying individuals; 150 non-Hispanic White American Male identifying individuals; and 150 Black/African American Male identifying individuals. For description of covariates see "Population characteristics" above.

Reporting for specific materials, systems and methods

We require information from authors about some types of materials, experimental systems and methods used in many studies. Here, indicate whether each material, system or method listed is relevant to your study. If you are not sure if a list item applies to your research, read the appropriate section before selecting a response.

Materials & experimental systems

n/a	Involved in the study
☒	☐ Antibodies
☒	☐ Eukaryotic cell lines
☒	☐ Palaeontology and archaeology
☒	☐ Animals and other organisms
☒	☐ Clinical data
☒	☐ Dual use research of concern

Methods

n/a	Involved in the study
☒	☐ ChIP-seq
☒	☐ Flow cytometry
☒	☐ MRI-based neuroimaging
